# Frailty and Macronutrients Intake Among Older Brazilian Adults

**DOI:** 10.1093/geroni/igab046.2998

**Published:** 2021-12-17

**Authors:** Carolina Freiria, Graziele Silva, Larissa Hara, Tábatta Brito, Flávia Arbex Silva Borim, Ligiana Corona

**Affiliations:** 1 University of Campinas, Campinas, Sao Paulo, Brazil; 2 University of Campinas, University of Campinas, Sao Paulo, Brazil; 3 Federal University of Alfenas, Federal University of Alfenas, Sao Paulo, Brazil

## Abstract

The adequate nutrition has an important role in the prevent and treatment of frailty, however, there are only few studies showing the relationship between macronutrients intake and this geriatric syndrome, especially in Latin countries. The aim of this study was to analyze the association between macronutrients intake and frailty among older adults in Brazil. This study included 521 community-dwelling individuals aged 60 years old or older. Frailty was assessed using a self-reported instrument and individuals were categorized in two groups: frail and non-frail (robust + pre frail). Food consumption was evaluated using the 24-hour recall and the software NDSR®. Differences between groups was assessed using the Mann Whitney test. The prevalence of frailty was 42.0%. Older adults considered frails presented lower intake of calories (1510.9 kcal vs 1639.3 kcal; p = 0.016), carbohydrates (196.8 g vs 213.3 g; p = 0.011), proteins (60.7 g vs 68.5 g; p = 0.016) and fiber (15.1 g vs 17.5 g; p= 0.002). They also had lower intake of protein per kilograms of weight (0.88 g/kg vs 0.99 g/kg; p= 0.010). The findings demonstrate high prevalence of frail in our sample, and that intake of most macronutrients was significantly lower among older adults with frail, indicating the importance of the screening of frail as well the evaluation of macronutrients intake among community-based older adults, to prevent malnutrition, sarcopenia and frailty in this population.

